# Proteoglycan Aggrecan Conducting T Cell Activation and Apoptosis in a Murine Model of Rheumatoid Arthritis

**DOI:** 10.1155/2014/942148

**Published:** 2014-01-29

**Authors:** A. Hanyecz, K. Olasz, O. Tarjanyi, P. Nemeth, K. Mikecz, T. T. Glant, F. Boldizsar

**Affiliations:** ^1^Department of Immunology and Biotechnology, Faculty of Medicine, University of Pecs, Szigeti u. 12, Pecs 7624, Hungary; ^2^Department of Medical Biology, University of Pecs, Szigeti u. 12, Pecs 7624, Hungary; ^3^Section of Molecular Medicine, Department of Orthopedic Surgery, Rush University Medical Center, 1735 W. Harrison Street, Chicago, IL 60612, USA

## Abstract

Rheumatoid arthritis (RA) is a systemic autoimmune disease and its targeting of the joints indicates the presence of a candidate autoantigen(s) in synovial joints. Patients with RA show immune responses in their peripheral blood to proteoglycan (PG) aggrecan. One of the most relevant animal models of RA appears to be proteoglycan-induced arthritis (PGIA), and CD4^+^ T cells seem to play a crucial role in the initiation of the disease. In this review, the role of various T cell epitopes of aggrecan in the induction of autoreactive T cell activation and arthritis is discussed. We pay special attention to two critically important arthritogenic epitopes, 5/4E8 and P135H, found in the G1 and G3 domains of PG aggrecan, respectively, in the induction of autoimmune arthritis. Finally, results obtained with the recently developed PG-specific TCR transgenic mice system showed that altered T cell apoptosis, the balance of activation, and apoptosis of autoreactive T cells are critical factors in the development of autoimmunity.

## 1. Structure and Function of the Cartilage Proteoglycan (PG) Aggrecan Molecule

PGs are complex macromolecules composed of a protein core to which glycosaminoglycan (GAG) and N-linked and O-linked oligosaccharide side chains are attached. The PG aggrecan (10–20% of the wet weight) provides a compressive strength to the articular cartilage. There are two major classes of PGs in articular cartilage: large aggregating PG monomers or aggrecans (henceforth PG aggrecan) and small PGs including decorin, biglycan, and fibromodulin [1]. They are synthesized by chondrocytes and secreted into the extracellular matrix, and their function is to maintain the fluid and electrolyte balance in the articular cartilage [2]. Most of the cartilage PG aggrecans are large molecules of high density which bind to hyaluronan (hyaluronic acid, HA) to form macromolecular aggregates [3–5]. Thus, the PG molecules do not exist in isolation within the extracellular matrix, rather they are present in aggregated form (PG aggregates). Each aggregate is composed of a central filament of HA to which up to 200 PG aggrecan molecules are bound, and each PG aggrecan-HA interaction is stabilized with a third component called link protein [6].

**Figure 1:**
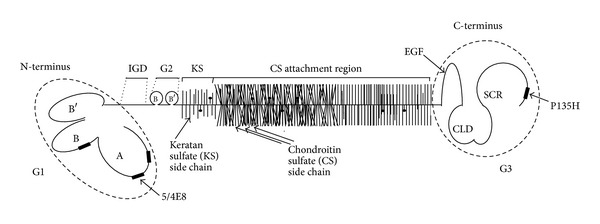
The schematic structure of PG aggrecan. The macromolecule consists of a central core protein to which hundreds of chondroitin sulphate (CS) and keratin sulfate (KS) side chains are attached. Note that the N- and C-terminal G1 and G3 domains are “overrepresented” in the figure for better visibility. The four most important T cell epitopes are indicated by black rectangles in the G1 and G3 domains. IGD: interglobular domain, SCR: complement regulatory protein-like module, and CLD: C-type lectin-like domain. Two dominant arthritogenic epitopes (5/4E8 and P135H), which are discussed in more detail in this work, are indicated.

The core protein of PG aggrecan contains three globular domains: two near the N-terminus (G1, which contains the HA-binding region, and G2) and one at the C-terminus (G3 domain) which contains epidermal growth factor-like, complement regulatory, and lectin-binding subdomains [7] (Figure 1). The G1 domain is composed of three functional subdomains termed as A, B, and B′, of which the B subdomain can bind to HA (Figure 1) [4, 6]. The G2 domain also possesses two B-type subdomains, but none of them can interact with HA, and, at present, their function is unknown. The G1 and G2 domains are separated by a short interglobular domain (IGD), and the G2 and G3 domains are separated by a long GAG-attachment region, which is rich in keratan sulphate (KS) and chondroitin sulphate (CS) side chains (Figure 1) [6].

The G3 domain resides at the carboxyl-terminus of the core protein and contains a variety of distinct structural domains (Figure 1) [6, 7]. This domain contains homology with the C-type lectin, but to date no distinct carbohydrate binding has been identified. It has been shown that PG aggrecan via this domain can interact with certain matrix proteins such as fibrillin, fibulins, or tenascin. These molecules can form a complex network. Therefore, a large number of PG aggrecan molecules form huge aggregates via its N-terminal G1 domain bound to HA and may interact with other macromolecules via their C-terminal G3 domain. In addition, the G3 domain is essential for normal posttranslational processing of the PG aggrecan core protein and subsequent secretion [8].

PG molecules rarely exist in intact form in the PG aggregates of the cartilage matrix, rather the PG aggrecan core proteins are subjected to proteolytic degradation. In arthritic diseases, cartilage undergoes irreversible destruction in response to various catabolic stimuli. Under such conditions, PG aggrecan molecules are known to be rapidly degraded and released from the cartilage matrix, followed by the degradation of matrix collagens. A number of matrix metalloproteinases (MMPs) and disintegrin and metalloproteinase with thrombospondin motifs (ADAMTS4/5) are the most dominant proteolytic enzymes which degrade PG aggrecan. These proteinases can cleave core protein of PG aggrecan at highly specific sites mostly located within the IGD [9–11]. Cleavage of the core protein results in the loss of the part of the PG aggrecan molecule bearing the KS- and CS-attachment domains, while the G1 domain remains attached to the HA filament and LP in the tissue. There are additional cleavage sites of the core protein of PG aggrecan, which contribute to the C-terminal truncation of PG aggrecan, the loss of the GAG chains, and the loss of tissue function [12].

## 2. Immunogenicity of Cartilage Components

Cartilage is one of the few immune privileged tissues in the body in which it is essentially avascular and therefore not subjected to close “internal” immunological surveillance. When it is degraded, however, unique antigenic molecules (neoepitopes) become exposed [15–17], released, and subsequently recognized by the immune system [18]. Thus, articular components may trigger and maintain immune responses to these antigens [19, 20].

Several autoantigens are described in RA, including a variety of proteins that become citrullinated in diseased joints. Humoral autoimmunity to citrullinated proteins in RA has gained increasing attention in recent years [21]. Citrullination is a posttranslational protein modification, where the amino acid arginine is converted to citrulline by peptidyl arginine deiminase-4 (PAD-4) [22, 23]. The presence of anticitrullinated protein Abs (ACPA) is highly specific for RA; ACPA are detected even in a higher proportion of patients than RF (although not all patients are positive) [24–27]. ACPA positivity is now included in the 2010 RA classification criteria [28]. The spectrum of ACPA-reactive proteins identified in RA patients so far includes citrullinated filaggrin [29–31], fibrinogen [32, 33], vimentin [34–37], type II collagen [33, 38–42], *α*-enolase [33, 43, 44], and some viral antigens [45–47].

An intriguing observation is that the appearance of ACPA in serum predates the onset of the clinical symptoms of RA [27, 48, 49]. An ACPA epitope mapping study found low titers of Abs recognizing only one epitope several years before disease onset, but both the epitope repertoire and the titers of ACPA increased markedly 2–4 years prior to the diagnosis of RA [49]. These observations clearly suggest that humoral autoimmunity to citrullinated proteins arises and expands via epitope spreading during the preclinical and clinical stages of RA.

The immune attack on the joints could also be initiated by a cross-reactive immune reaction in response to unrelated antigens by the mechanism of “molecular mimicry.” The net result of such autoimmune reactions is that cartilage is degraded further and more autoantigens are released. This may lead to a chronic, self-perpetuating inflammation in genetically predisposed individuals who are prone to develop these autoimmune reactions [50]. Although autoimmunity to cartilage proteoglycans has been studied less intensively than autoimmunity to type II collagen, cartilage PG (aggrecan) is considered as causal/contributing factor in rheumatoid joint disease [2, 13, 51, 51–58].

Patients with rheumatoid arthritis (RA) show cellular immune responses in their peripheral blood to PG aggrecan [2, 54]. The incidence of immune response to PG aggrecan epitopes in patients with RA varied from 5 to 85%, depending upon the study. Cellular immunity to proteoglycan has also been described in patients with juvenile rheumatoid arthritis [54], and immunoreactive fragments of PG aggrecan and anti-PG antibodies have been demonstrated in the synovial fluids of patients with RA [59]. All these studies suggest that cartilage PG aggrecan may play an important role in the development of autoimmunity against peripheral joints.

## 3. Rheumatoid Arthritis (RA) and Its Experimental Animal Models

RA is one of the most frequent systemic autoimmune diseases; it affects approximately 1% of the human population with a female preponderance. Genetic factors play a significant role in RA susceptibility. However, the concordance of this disease in monozygotic twins is only 15%. It has been therefore hypothesized that, while RA susceptibility is determined genetically, disease onset may depend on nongenetic (i.e., environmental), epigenetic, or posttranslational events [60].

Several lines of evidence indicate that the effector mechanism, which initially attacks small joints, is T cell driven. As a result, an aggressive synovial pannus develops, which destroys articular cartilage and bone, leading to massive ankylosis and deformities of peripheral joints. The disease has a progressive character, with the involvement of more and more joints. Although RA is a systemic autoimmune disease, it targets peripheral joints which suggests the presence of candidate autoantigen(s) in synovial joints [2, 8, 61]. There are numerous rodent models that simulate some or many of the clinical, immunological, or histopathological features of the disease. It has become a strong working hypothesis that MHC and non-MHC genetic components share loci that are common in various autoimmune diseases and in corresponding animal models [62]. The most relevant animal models of rheumatoid arthritis appear to be those induced by cartilage matrix components such as PG aggrecan or type II collagen [63–65]. The pathologic basis in both model systems appears to be cross-reactive immune reactions: T cells and antibodies raised against the immunizing heterogeneous (bovine, human) cartilage antigens recognize and subsequently attack the mouse's own tissues (self) [66, 67].

## 4. PG (Aggrecan)-Induced Arthritis (PGIA)

Systemic immunization of genetically susceptible strains of mice with human cartilage PG that has been depleted of GAG side chains, that is, more or less degraded, leads to the development of progressive polyarthritis [8, 63]. PGIA shows many similarities to RA as indicated by findings of clinical assessments, radiographic analyses, scintigraphic bone scans, laboratory tests, and histopathologic studies of synovial joints [8, 63].

PGIA was first described in the BALB/c strain [63], but certain C3H colonies (e.g., C3H/HeJCr) were also found to be susceptible to PGIA [68]. During the immunization protocol, female mice are injected intraperitoneally with 100 *μ*g of cartilage PG aggrecan (measured as protein) in adjuvant (CFA/IFA or DDA) three-times every third week. The initial clinical manifestations of joint inflammation (swelling and redness) appear after the third to fourth intraperitoneal injection depending on the source of PG antigen, adjuvant, and BALB/c or C3H colony used [4, 63, 66, 68–70]. Joint inflammation starts as polyarticular synovitis in small peripheral joints. During the early phase, lymphocytes and polymorphonuclear leukocytes invade the synovium. This is followed by gross, “tumor-like” proliferation of synovial lining cells and fibroblasts. Once an animal develops, arthritis repeated “spontaneous” episodes of inflammation result in complete destruction of the articular cartilage and erosion of the subchondral bone, which leads to severe deformities of the peripheral joints [63, 66, 69]. The disease is polygenic, has a recessive inheritance, and major histocompatibility complex (MHC) and non-MHC components are critical for the development of arthritis [71–73].

PGIA in BALB/c mice is a T cell-dependent and (auto)antibody/B cell-driven disease. Antibodies against immunizing (human) PG aggrecan appear during the second/third week of immunization. T cell response to PG aggrecan is detectable approximately 5–7 weeks after the first immunization, and along the course of the disease the humoral and cellular immune responses slowly decline as the disease becomes chronic and less active [63, 66, 69].

CD4^+^ T cells have been implicated in the development of PGIA by observations that anti-CD4 mAb treatment prevents arthritis and that the transfer of the disease requires T cells from arthritic animals [74, 75]. BALB/c mice are genetically predisposed to a Th2-type immune response; however, immunization of these mice with PG aggrecan induces a higher ratio of IFN-*γ* to IL-4, indicating that PGIA is a Th1-type disease and immunization with PG aggrecan in either Freund's adjuvant or DDA is a sufficient Th1 stimulus to overcome the genetic inclination toward development of a Th2-type response [76].

Treatment with Th2 cytokines before the onset of arthritis prevents the development of arthritis, indicating that a switch from a Th1-type response to a Th2-type response is critical for the control of joint inflammation associated with arthritis [76]. Moreover, in PGIA, neutralization of IFN-*γ* inhibits arthritis and IFN-*γ* ^−/−^ mice develop arthritis with delayed onset and reduced severity in comparison to wild-type (WT) mice. These findings indicate that IFN-*γ* is an important proinflammatory cytokine promoting disease severity in PGIA [76].

IL-17 has emerged as an important proinflammatory T cell cytokine in several models of arthritis [77–79]. In line with this, significant amount of IL-17 production was detected in the early (initiation) phase of PGIA in normal BALB/c mice [80]. IL-17 production by isolated peritoneal- (from the site of PG immunization) or spleen cells was detected already after the first and second antigen (PG) challenges, before the development of the clinical signs of arthritis, showing that the Th17 cells were generated both locally and systemically [80]. Based on these results we proposed a pathogenic role for IL-17 in PGIA initiation [80]. However, contrary to the dependence on IL-17 described in other models of arthritis (e.g., collagen-induced arthritis), IL-17-deficient mice developed PGIA similar to WT animals [81]. This contradiction was resolved when IL-17/IFN*γ* double knockout mice were tested for PGIA [82]. Results from this Th1/Th17 deficient system have clearly shown that the IL-17 “independency,” found earlier, was due to a more robust IFN-*γ* response in the absence of IL-17 which compensated the effect of IL-17-deficiency [82]. When IFN-*γ* was absent too, the effect of IL-17 could be observed more clearly in PGIA [82]. Taken together, these data suggest that PGIA is a disease with Th1 dominance; however, contribution from IL-17 (similar to human RA), especially in the initiation of the autoimmune reactions, has to be considered too [80].

## 5. Collagen-Induced Arthritis (CIA)

Besides PGIA, collagen-induced arthritis (CIA) is the other widely used experimental autoimmune arthritis model system. CIA was originally elicited in rats following a single intradermal injection of type II collagen (CII) emulsified in Freund's adjuvant [64]. Further studies demonstrated that a similar pathology could also be induced in primates [83] and in susceptible strains of mice [65]. CIA can be induced using native autologous or heterologous CII and is specific to CII, since immunization with type I or III collagen failed to induce disease [64, 65]. While either incomplete (IFA) or complete Freund's adjuvant (CFA) can be used to trigger CIA in rats, the induction of disease in mice generally requires the presence of heat-killed mycobacterium tuberculosis in CFA [65]. Immunization with CII/CFA results in a rapid and severe polyarthritis of the peripheral articular joints which first appears around 3-4 weeks after antigen challenge and becomes progressively worse for approximately 2–4 weeks before slowly waning. Whilst the pathology is similar when CIA is induced with either autologous or heterologous CII, the nature of the disease differs; autologous CII induces a more chronic disease with a delayed onset and reduced penetrance [84, 85]. In both cases the histopathology of inflammatory arthritis resembles human rheumatoid arthritis (RA). Like RA, CIA is characterized by the presence of fibrin deposition, hyperplasia of synovial cells, periosteal bone formation, mononuclear infiltrates, pannus formation, and eventual ankylosis of one or more articular joints [64].

While the precise mechanisms by which immunization with heterologous or autologous CII in CFA leads to a chronic arthritis in susceptible mice are not known, there are considerable data to implicate CII-reactive CD4^+^ T cells as the primary mediators of disease induction and complement-fixing anti-CII autoantibody production by B cells as the major immune mechanism leading to the localized chronic inflammatory response [86]. CIA is classified as a Th1-mediated disease based on the abundant IFN-*γ* production [87]. However the role of IFN-*γ* in CIA is more complex. Complete elimination of IFN-*γ* or IFN-*γ* receptor signaling led to the exacerbation of disease [88, 89]. On the other hand, neutralization of IFN-*γ* at an early stage of disease inhibited arthritis [90]. The ability of IFN-*γ* to suppress Th17 cells appears to account for augmented disease in IFN-*γ* ^−/−^ or IFN-*γ* receptor deficient mice in CIA, as inhibition of IL-17 with neutralizing antibodies suppressed arthritis [91]. Autoreactivity to cartilage CII in human RA patients, although not a defining feature of the disease, has been clearly demonstrated [92, 93].

## 6. T Cell Epitopes in Cartilage PG Aggrecan 

Previously, in an epitope mapping study using a total of 143 synthetic peptides containing predicted T cell epitopes, 27 peptide sequences were identified wich induced T cell responses in PG-immunized BALB/c mice [4]. An epitope hierarchy, accounting for the different effector functions of PG (aggrecan)-specific T cells, and determinant spreading, has been established. Some of the T cell epitopes were full T cell activators, whereas a number of subdominant and cryptic epitopes proved to be partial activators *in vitro*, inducing either cytokine secretion or T cell proliferation, but not both [4]. A few T cell epitopes of the core protein of cartilage PG aggrecan were clearly recognized by T cells in PG-immunized arthritic animals, but the corresponding peptides did not induce T cell responses when injected into naive BALB/c mice; thus these T cell epitopes were designated as “conditionally immunogenic” [4].

Importantly, T cell responses to only four epitopes were clearly associated with arthritis induction in mice (henceforth “arthritogenic” epitopes) [94]. Out of these four epitopes, three were found in the G1 and one in the G3 domain of PG aggrecan (Figure 1) underlining the importance of these regions in the induction of arthritis. More specifically, P49–63 and P70–84 lie in the A loop, whereas P155–169 lies in the B loop of the G1 domain and P2373–2387 in the G3 domain of PG (Figure 1) [94]. The positions of arthritogenic T cell epitopes clearly show that the two terminal regions of the large PG molecule are involved in the induction of T cell response, most likely because they are more easily accessible to the immune system than the inner regions which are “covered” with KS and CS side chains (Figure 1). Two T cell hybridomas (5/4E8 and P135H), established earlier from the T cells of mice with PGIA, also recognized two of the above-mentioned arthritogenic epitopes: P70–84 and P2373–2387, respectively [95, 96]. Injection of either unprimed or peptide-primed 5/4E8 or P135H hybridomas into naive BALB/c or BALB/c^SCID^ mice, respectively, induced arthritis. These observations underline the importance of the G1 and G3 domains in PG in the generation of arthritogenic T cells.

## 7. The Hypothetic Role of T Cells Specific for a “Shared Epitope-Like” Sequence in the G3 Domain of PG, in the Initiation of Autoimmune Inflammation in PG-Induced Arthritis

The G3 domain resides at the carboxyl-terminus of the core protein of PG aggrecan and is structurally distinct from the G1 and G2 domains. It contains an EGF module (EGF1), a calcium-binding EGF module (EGF2), a C-type lectin module (CLD), and a complement regulatory protein-like module (SCR) (Figure 1). The C-type lectin module is constitutively expressed and mediates binding to other extracellular matrix molecules, for example, tenascin-R, fibulin-1, fibulin-2, and fibrillin-1 [8]. The subdomains (modules) of the G3 domain are subjected to alternative splicing. The expression of the SCR module is variable, but this module is usually present in humans regardless of the age. The EGF1 and EGF2 modules are expressed to a lower extent (25–28% and 5–8%, resp., in humans). The EGF2 module (a splice variant) is highly conserved and uniformly expressed at low levels in several species, whereas the less conserved EGF1 module is expressed at different degrees due to also a splicing mechanism [7, 97]. These differences and the alternative splicing of the EGF-like repeats may reflect different functions for the EGF1 and EGF2 modules in different species. Expression of the EGF modules could constitute a mechanism for feedback regulation of differentiation and proliferation of chondrocytes. In addition, alternatively spliced modules of the G3 domain could affect GAG substitution of PG and transport through the secretory pathway. The alternative splicing of the flanking EGF and SCR modules could also have a regulatory function by modulating the C-type lectin-mediated interactions [8]. The loss of PG aggrecan is a major feature of cartilage degradation associated with arthritis [98]. There is an age-related loss of the G3 domain of PG, and 92% of the G3 domain is lost as part of the normal turnover of the PGs, whereas the rest of the molecule bound to HA is retained in the cartilage [99].

As described in the previous section, while most of the T cell epitopes are located within the poorly glycosylated globular G1 domain [94], one of the epitopes is present in the G3 domain at the C-terminus of the human PG. Hyper-immunization of BALB/c mice, with a synthetic peptide (p135H) representing a segment of the human PG G3 sequence (^2373^TTYKRRLQKRSSRHP) followed by the injection of a single dose of PG, induced progressive polyarthritis [100]. Using an adoptive transfer system, p135H peptide-stimulated lymphocytes and T cell hybridomas from peptide p135H-primed BALB/c mice were also capable of inducing arthritis in SCID mice “presensitized” with a single injection of PG aggrecan, with a relatively high incidence and severity score [100].

**Figure 2:**
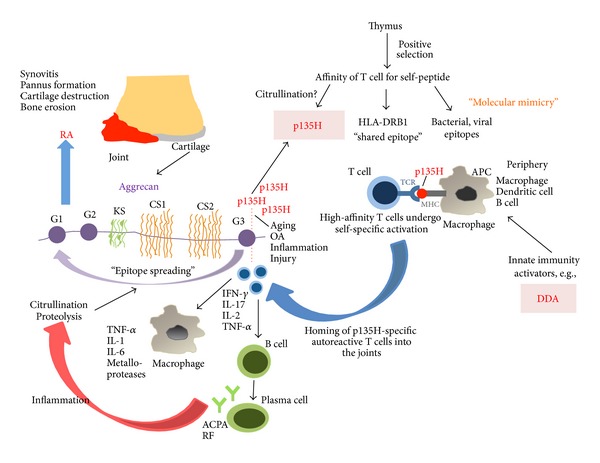
Potential role of the P135 epitope found in the G3 domain of PG aggrecan in the development of arthritis. Importantly, this epitope shares a significant sequence homology with the HLA-DRB1 “shared epitope” sequence (QKRSS in p135 versus QKRAA in HLA). Age-related release of the G3 domain of PG aggrecan could lead to the activation and differentiation of autoreactive T cells. T cell tolerance could be broken by several factors including neoepitope formation (e.g., citrullination) or molecular mimicry mechanisms (for more details see Section 6). Activated P135-specific T cells could “home” into the joints, where further activation would lead to cytokine release and the activation of B cells and macrophages. The initiation of the joint inflammation could perpetuate PG aggrecan degradation; the proteolysis and increased citrullination could pave the way for “epitope spreading.” The involvement of the G1 domain contributes to the activation of even more autoreactive T cells taking the autoimmune attack of the joints into a final irreversible stage.

A special feature of this T cell epitope is that the p135H contains a conserved amino acid sequence QKRSS, which shows striking similarity to the “shared epitope” (SE) of QKRAA amino acid sequence. This SE is located in the third hypervariable region of certain HLA alleles associated with RA [101]. HLA-derived peptides encompassing the SE sequence can randomly select T cells that bind the self-derived peptide at low avidity. Later in life, these previously quiescent QKRAA-specific T cells can be activated by binding with high avidity an exogenous peptide containing the SE [101]. Interestingly, proteins in common human pathogens, such as *Escherichia coli* (DnaJ heat-shock protein), *Lactobacillus lactis*, *Brucella ovis* (DnaJ heat-shock protein), Epstein-Barr virus (gp110 protein), and human JC polyomavirus (capsid protein VP3) have been identified wich express the SE in the context of highly immunogenic proteins, and immune responses to several of these antigens have been evaluated in RA patients (Figure 2) [102–105]. These sequence data suggest that immunologic cross-reactivity might exist between certain RA-associated HLA alleles, the above-mentioned immunogenic proteins of human pathogens and the p135H peptide sequence—a T cell epitope of human cartilage PG aggrecan. Intermittent exposure of the immune system to these bacterial or viral antigens at mucosal surfaces might lead to the activation of resting QKRAA-specific T cells and then the T cell activation is perpetuated by encounters with peptides of self-origin, encompassing the SE sequence (Figure 2). It seems to be an attractive hypothesis that the sequence of p135H in human cartilage PG aggrecan can serve as an analog of SE, and it may be responsible for the joint-specific homing of QKRAA-reactive T lymphocytes and then be involved in the initiation of the autoimmune process in RA (Figure 2) [101].

As described above, in the process of aging, the vast majority of the G3 domain is lost as the part of the normal turnover of cartilage PG aggrecan [99], whereas the rest of the molecule, including the G1 domain which is bound to hyaluronan, is retained in the cartilage. The C-terminal tail of PG is cleaved first, and two tandem “boxes” (RRXXK and RXXR) of the consensus sequences have been demonstrated to be involved in the earliest cleavage [6]. Interestingly, both cleavage sites described are located within p135H peptide sequence (TTYKRRXXKRXXRHP). Therefore, a significant amount of p135H might be released from articular cartilage due to normal turnover or due to an enhanced proteolytic processing of the G3 domain (e.g., in inflammation or in cartilage injury) and then be exposed to the immune system in the joints (Figure 2). We might also suggest that this C-terminal part of the G3 domain plays a role in the initial activation of T cells and, later, the rest of the molecule, especially the G1 domain, becomes the main target. This process can be interpreted as epitope spreading, that is, in the course of the autoimmune inflammation when neo-epitopes are generated (e.g., by citrullination of other parts of the molecule or by enhanced proteolytic activity), which subsequently become additional targets for the autoimmune response (Figure 2).

Unlike T cells, which react with epitopes in the G1 and G3 domains of PG aggrecan, B cell epitopes are mostly found in the CS attachment region of the molecule (Figure 1) [4]. Partial deglycosilation of aggrecan is required for a successful induction of PGIA, because the remaining CS “stab-clusters” provoke a strong antibody response [4]. Besides the age-related loss of the G3 domain, the partial loss of CS side chains is also a common change in PG aggrecan, leading to the “uncovering” of important B cell epitopes. Reactive B cells have strong antigen presenting capacity and thus might contribute to the initial activation of T cells (Figure 2) [4, 106]. Activated T cells, in turn, help plasma cell differentiation leading, eventually, to the production of aggrecan-specific antibodies (Figure 2) [106].

## 8. The G1 Domain

The G1 domain contains an immunoglobulin-like A loop and HA-binding B and B′ loops (Figure 1) [94, 107]. As mentioned above, three dominant/arthritogenic and four subdominant epitopes are located in the G1 domain and only a few cryptic or subdominant epitopes are located in the other regions of the PG molecule. Thus, only <0.02% of the complete molecular mass, or <15% of the core protein, drives the arthritogenic response to PG in genetically susceptible BALB/c mice.

For the further characterization of T cell epitopes recognized in the PG molecule, a PG (aggrecan)-specific V*β*4^+^/V*α*1^+^ Th1 hybridoma (5/4E8) was isolated from mice with PGIA. This CD3^+^/CD4^+^ T cell hybridoma induced inflammatory processes in the joints when injected intravenously into naive irradiated BALB/c mice. I-A^d^ restricted recognition of PG induced IL-2 and IFN-*γ* secretion and the expression of CD44, CD28 and LFA-1 [95, 108]. The clinical symptoms and histopathological features of this 5/4E8 T cell hybridoma-induced arthritis was very similar to that described in transfer experiments using lymphocytes from arthritic animals [74, 109], but the onset of arthritis was earlier and the incidence ranged between 45 and 90% [95]. The 5/4E8 epitope (^70^ATEGRVRVNSAYQDK^84^; the core sequence is underlined) is located on the G1 domain of human PG molecule (Figure 1) [4] and has been identified as a dominant and probably the most arthritogenic T cell epitope of PG in the genetically susceptible BALB/c strain [94, 96]. A peptide fragment of the G1 domain of PG aggrecan, containing the 5/4E8 epitope, was able to activate T cells derived from human patients with RA [56]. Most recently, two independent laboratories have reported that a citrullinated version of the 5/4E8 epitope-containing aggrecan peptide VVLLV***ATEGR/CitVRVNSAY*** *QDK* (5/4E8 sequence bold-faced in italics and arginine/citrulline [R/Cit] substitution underlined) induced substantial cytokine (IL-17, IL-22, IL-6, TNF*α*, and IFN*γ*) production by T cells from the majority of RA patients [58, 110]. RA T cells responded poorly to the native (noncitrullinated) peptide in both studies, and T cells from healthy subjects did not respond [58] or responded only to the citrullinated peptide by producing IL-6 [110]. Although the majority of RA patients tested were ACPA^+^ (anti-CCP^+^), T cell response to the citrullinated peptide was also noted in some ACPA^−^ patients [58, 110]. These results suggested that the 5/4E8 epitope (either native or citrullinated form) plays a crucial role in PGIA and possibly involved in RA.

The importance of the G1 domain in the development of autoimmunity is clearly shown in a recent modification of the PGIA model; immunization with the recombinant human G1 (rhG1) domain could also trigger autoimmune arthritis (henceforth G1 domain-induced arthritis “GIA” model) in BALB/c mice [111]. The clinical phenotype, histopathological abnormalities, and laboratory test results in the GIA model were very similar to those described in “parental” PGIA, but side-by-side experiments showed that mice with GIA developed arthritis more uniformly and with higher overall inflammation scores than those with PGIA. The incidence of arthritis reached 95–100% in both models, although disease severity was higher in the GIA than in the PGIA model. T cells from mice with GIA produced more IL-2 and were activated on a higher level upon *in vitro* stimulation with rhG1 than with PG; likewise, T cells from mice with PGIA responded more robustly to stimulation with PG than with rhG1. Autoantibodies against self (mouse)-PG were produced in same amounts in both PGIA and GIA. When disease-associated cytokines and antibodies were compared, higher serum levels of IL-1*β*, IL-17, and IgG-type RF and a higher IgG2a : IgG1 ratio of antimouse PG autoantibodies, were detected in GIA, but higher quantities of anti-CCP antibodies (ACPA) were measured in the PGIA model. PGIA is known as a Th1-type of the disease with a strong IFN-*γ* dominance [82] associated with significant IL-17 production [80]. A more pronounced production of IL-17 was found in GIA suggesting that the latter disease is rather a mixed Th1/Th17-mediated form. The detected differences between the IL-17 and IFN-*γ* production could contribute to the more severe clinical symptoms of arthritis in mice with GIA. Because the two models (PGIA and GIA) could be distinguished on the basis of “RA-specific” serologic markers (RF and ACPA), they may represent two subtypes of seropositive RA [111].

## 9. T Cell Receptor (TCR) Signaling and Apoptosis in Arthritis

So far, we have reviewed the most important T cell epitopes present in the PG aggrecan molecule, which could contribute to the induction of autoimmune T cell activation and differentiation in animal models and perhaps in RA. Importantly, T cell tolerance towards self-antigens is maintained by several parallel mechanisms. Thymic negative selection resulting in “central” tolerance is completed by peripheral tolerance mechanisms including regulatory T cells, suppressive cytokines, and inhibitory costimulatory signals. When any of these mechanisms of tolerance is broken by internal or external factors, autoreactive T cells may accumulate and pave the way to autoimmune inflammatory processes.

An important step to activate such events is the recognition of peptides bound to MHC molecules of antigen presenting cells (APCs), which initiates a cascade of intracellular signaling events in T cells. TCR signaling proceeds through a number of well-known steps, including the activation of Lck by CD45 and then the phosphorylation of ZAP-70, followed by phosphorylation of LAT, SLP-76, and PLC*γ* [112]. Later, the intracellular Ca^2+^ level rises, the MAPK cascade is also activated, and the net effect is the activation of some important transcription factors (AP-1, NF-AT, and NF-*κ*B). As a consequence, activated T cells will enter the cell cycle, produce cytokines, and differentiate. Normally, the activation of T cells is limited by activation-induced cell death (AICD). However, several lines of evidence show that dysregulation of TCR signaling, activation, and apoptosis could play a role in the pathogenesis of RA.

TCR transgenic (TCR-Tg) mice were also generated, in which mice more than 90% of the CD4^+^ T cells expressed the V*α*1.1 and V*β*4 chains recognizing dominant and possibly most arthritogenic “5/4E8” T cell epitope (see above in Section 7) [4, 113]. Importantly, this transgenic mouse offered an excellent tool to study the above-mentioned TCR signaling events in the context of PGIA.

**Figure 3:**
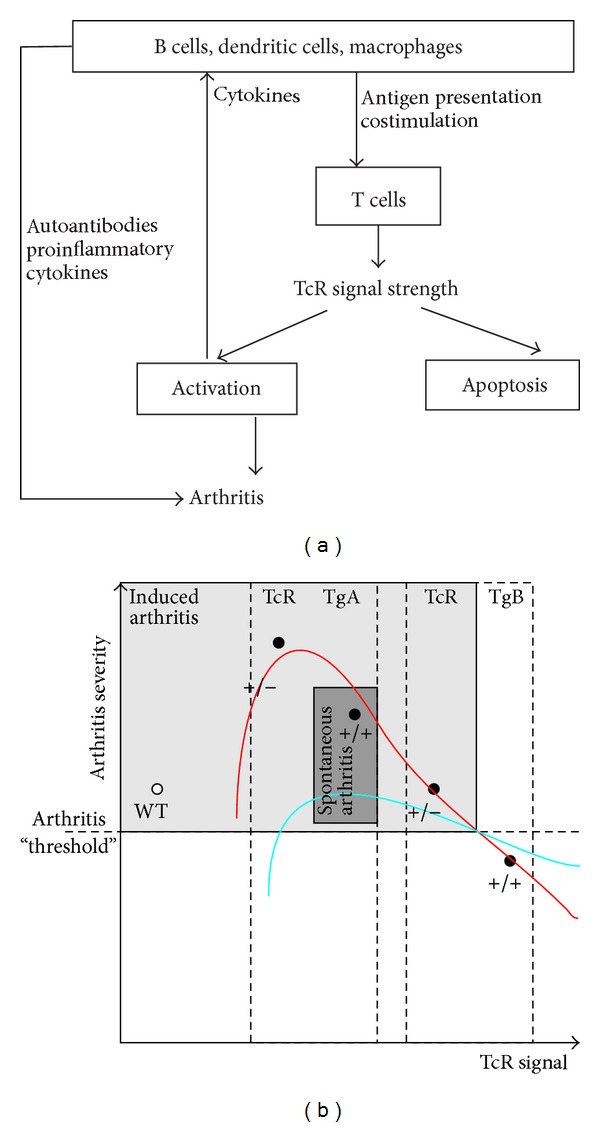
The balance of T cell activation and apoptosis regulates the development of autoimmune arthritis in PGIA. (a) The interaction between APCs and T cells determines T cell activation. TCR signal strength is regulated by costimulation and TCR expression. When the activation signal is optimal, T cell activation will lead to the autoimmune attack of joints. (b) Relation between the strength of the TCR signal and arthritis severity in the PG-specific TcR-Tg mice. The two mouse colonies (TCR-TgA and TCR-TgB) were described in [13]. The use of homo- (+/+) or heterozygous (+/−) TCR-Tg mice allowed us to further refine our TCR signal strength studies in PGIA. Activation (red line) or apoptosis (blue line) of T cells is regulated by TCR signal strength. When the activation exceeds apoptosis, the autoreactive cells accumulate and arthritis develops (grey area). When apoptosis overrides activation the autoreactive T cells are eliminated and arthritis does not develop. Spontaneous arthritis (dark grey zone) was only observed in homozygous TCR-TgA mice [14]; thus, at least from the T cell signaling side, a very narrow “window” exists when T cell activation is optimal and is over the apoptosis.

Two transgenic (TCR-TgA and TCR-TgB) lines were generated from different pronuclear injections using the same construct [114]. Transgene-positive founders were backcrossed into the BALB/c background. Both TCR-TgA and TCR-TgB strains possess the same epitope (5/4E8)-specific TCR expressing CD4^+^ T cell repertoire. However, CD4^+^ T cells of TCR-TgB mice expressed twice as much TCR on their surface when compared to the TCR-TgA line [114]. Contradictory to the level of TCR expression (higher in TCR-TgB mice), TCR-TgA mice developed earlier a very severe arthritis when immunized with either PG or rhG1 compared to the WT BALB/c controls [114], and, surprisingly, TCR-TgB mice exhibited delayed onset and less severe arthritis than TCR-TgA mice [114]. When the underlying mechanisms of these profound differences were investigated, elevated serum levels of anti-G1 antibodies were found in the TCR-TgA strain before the first visible signs of inflammation appeared [114]. Concordant with the higher serum levels of G1-specific antibodies, a higher number of antibody producing B cells was found in TCR-TgA mice [114]. Secretion of these antigen-specific antibodies is not only part of the B cell function, because these B cells are very important as APCs in PGIA [4, 106, 115, 116] and probably in GIA as well (Figure 3(a)). The higher number of B cells in TCR-TgA mice could present antigen to T cells more effectively and led to a more extensive T cell activation (Figure 3(a)). In turn, activated CD4^+^ T cells play key role in the initiation of PGIA and GIA (Figure 3(a)) [80, 111]. Furthermore, fine phenotypic analysis of TCR-TgB mice revealed a lower percentage of ICOS^high^ CD4^+^ T cells, probably follicular T helper cells (Tfh), which are critical in systemic autoimmunity by supporting autoreactive B cells [117]. ICOS has been shown to be indispensable in collagen-induced and K/BxN arthritis models [118–120], and certain ICOS polymorphisms are associated with RA [121]. The importance of costimulatory signals in RA (Figure 3(a)) is also supported by the successful clinical use of Abatacept, a recombinant fusion protein of CTLA-4 [122].

As described above, the differential expression level of the TCR in the TCR-TgA and -TgB mice was contrary to the clinical phenotype; the higher TCR expression led to less severe arthritis [114]. In the background, a significantly stronger TCR signal (higher phosphorylation levels of key signaling molecules, e.g., ZAP-70 and p38) was found in the TCR-TgB mice, which expressed twice as much TCR on the surface of CD4^+^ T cells when compared to the TCR-TgA line [114]. The higher TCR expression and stronger T cell signaling provoked extensive apoptosis of CD4^+^ autoreactive T cells during immunization with an antigen (rhG1) containing the 5/4E8 epitope. This might lead to the elimination of activated arthritogenic T cells and decreased arthritis severity (Figure 3(b)) [114]. All of these apoptosis studies have been performed using the AnnexinV/PI staining and analyzed by flow cytometry. Further studies would be needed to determine which apoptosis pathways are activated.

Altered T cell apoptosis has been found as a critical factor in the development of autoimmune arthritis by others as well [14, 123, 124]. Results obtained with the PG-specific TCR-Tg mice showed that TCR signal strength controls the onset and severity of arthritis by regulating the activation and apoptosis of T cells (Figure 3(b)). An optimal TCR signal leads to a strong activation of T cells which then induce arthritis very efficiently leading to a “superarthritic” phenotype, while an extremely “strong” TCR signal generated only a milder form of arthritis (Figure 3(b)). Thus, the balance of activation and apoptosis of autoreactive T cells (i.e., arthritis severity) seems to be controlled by TCR signal strength (Figure 3(b)).

In aging homozygous TCR TgA mice, arthritis developed spontaneously, too [14]. Although the clinical picture was somewhat different from the PGIA, we hypothesize that this can also be explained by TCR signal strength as described above (Figure 3(b)). A low threshold, continuous activation of autoreactive T cells associated with impaired apoptosis may result in the development of spontaneous arthritis [14] (Figure 3(b)). Spontaneous arthritis never developed in the TCR TgB strain, most likely to the stronger TCR signal, which led to the continuous elimination of the autoreactive T cells (Figure 3(b)). This, again, supports our hypothesis about the regulatory role of TCR signal strength in autoimmune arthritis (Figure 3).

## 10. Concluding Remarks

Herein we summarized our current knowledge of PG immunity mostly based on the results of PGIA model with special attention to T cell activation. This mouse arthritis model has provided profound insight not only to the most important T cell epitopes of PG aggrecan but also to their role in T cell activation and apoptosis. Based on data from recently developed TCR-Tg mice, a new hypothetic model could be established showing the importance of TCR signal strength in the pathomechanism of arthritis. Although PG aggrecan is only one of the potential autoantigens in cartilage or joint, which can drive the autoimmune attack in RA, there is increasing evidence that this tissue-restricted cartilage macromolecule (PG aggrecan) may also be a potential autoantigen in RA and some analogies can be found in the mechanism of PGIA and RA.
